# Global athlete mental health screening: cross-cultural validity of the athlete psychological strain questionnaire

**DOI:** 10.3389/fpsyg.2026.1761749

**Published:** 2026-03-12

**Authors:** Teodora-Simina Dragoiu, Florentina Ligia Furtunescu, Adela Caramoci, Alina Maria Stancu-Smaranda, Oliver R. Runswick

**Affiliations:** 1 Department of Public Health and Management, Carol Davila University of Medicine and Pharmacy, Bucharest, Romania; 2 Department of Sports Medicine, Carol Davila University of Medicine and Pharmacy, Bucharest, Romania; 3 Department of Psychology, Institute of Psychiatry, Psychology and Neuroscience, King’s College London, London, United Kingdom

**Keywords:** APSQ, cross-cultural validity, mental health screening, questionnaire, SMHAT-1, sports psychology, validation

## Abstract

**Introduction:**

Mental health screening is important across sports cultures, yet recommended tools primarily exist in English. Several translations of the Sport Mental Health Assessment Tool-1 have been published and supported by validation studies; however, it is the Athlete Psychological Strain Questionnaire (APSQ) score that determines whether athletes should continue with the screening process.

**Objectives:**

We aimed to investigate the validity of the Romanian APSQ within a representative population and examined the associations between the APSQ score and a range of well-being measures.

**Methods:**

206 Romanian-speaking elite athletes from Romania’s national and Olympic teams completed an online questionnaire that included demographic and relevant questions, followed by the APSQ-Ro, the second step of SMHAT-1, and the WHO-5 Well-Being Index.

**Results:**

The APSQ-Ro demonstrated good internal consistency, suitable parameters for factorial analysis, a one-factor model with acceptable fit indices, and significant divergent validity with the WHO-5. The APSQ cut-off demonstrated a good sensitivity for depression, anxiety, and sleep disturbances, though with low specificity for all mental health problems assessed during SMHAT’s second step. When analysing the range of APSQ values, good diagnostic ability (as measured by the area under the curve values) was detected for anxiety and depression. In contrast, poor overall AUC values and AUC at threshold were both identified for drug use and disordered eating.

**Conclusion:**

The limited diagnostic capacity for certain problems and the non-significant correlation between the explored variables may be attributed to several factors, including cultural influences.

## 1 Introduction

The mental health of elite athletes is vital to health and performance, and is becoming central to Sports Medicine research (Reardon et al., 2019; Chang et al., 2020; Stillman et al., 2019; Gwyther et al., 2024). Recent findings show that the athletic population is vulnerable to common mental health problems seen in the general population, and additionally, they experience sport-specific stressors (Reardon et al., 2019; Stillman et al., 2019; Gwyther et al., 2024; Bär and Markser, 2013; Rice et al., 2016). Mental health issues such as depression, generalised anxiety disorder, and sleep problems have shown varying prevalence rates across studies (Reardon et al., 2019; Gwyther et al., 2024; Oevreboe et al., 2023; Van Slingerland et al., 2019). Some, such as eating disorders, are more frequent in the athletic population compared to the general population (Reardon et al., 2019; Åkesdotter et al., 2020; Mountjoy et al., 2023). Various risk factors, including general characteristics such as female gender (Reardon et al., 2019; Gwyther et al., 2024; Rice et al., 2016) age and perfectionism (Gwyther et al., 2024) or sport-specific factors, such as individual sports (Bär and Markser, 2013; Gorczynski et al., 2024; Pluhar et al., 2019), increased number of injuries (Reardon et al., 2019; Rice et al., 2016), or aesthetic sports (Gwyther et al., 2024; Bär and Markser, 2013; Rice et al., 2016), are discussed in multiple studies. Most of the research focused on prevalence data and risk factors, with some addressing the screening and management of mental health problems (Chang et al., 2020; Gouttebarge et al., 2021; Moesch et al., 2018). Early detection of mental health problems through regular and efficient mental health screening is of utmost significance to provide adequate support and prevent negative consequences, but there is a lack of tools available in some languages, and little is known about how cultural factors influence screening processes (Anderson et al., 2025).

A range of screening methods using self-assessment questionnaires have been described in the general psychiatric literature, such as the Patient Health Questionnaire-9, Kessler Distress Questionnaire 10 or the Generalised Anxiety Disorder 7 (Gouttebarge et al., 2021; Kessler et al., 2002; Kroenke et al., 2001; Spitzer et al., 2006). Still, these may not capture the specific stressors and nature of mental health in athletes. As a result, athlete-specific mental health screening questionnaires have been developed, such as the Athlete Sleep Satisfaction Questionnaire, the Baron Depression Screener for Athletes and the Brief Eating Disorder in Athletes Questionnaire (Reardon et al., 2019; Gouttebarge et al., 2021; Bender et al., 2018; Driller et al., 2018; Martinsen et al., 2014; Baron et al., 2013). In addition, questionnaires covering broader mental health screening rather than specific problems were developed, including the Athlete Psychological Strain Questionnaire (Rice et al., 2020) that can be used as an initial screen, the Athlete Psychological Well-Being Inventory (Santi et al., 2024), that can be applied in the context of sport-related injuries, and the Mental Health Screening Instrument for Athletes, initially validated in a sample of collegiate athletes (Donohue et al., 2023). In 2020, the International Olympic Committee published a consensus statement describing a new mental health screening tool: the Sport Mental Health Assessment Tool 1 (SMHAT-1) (Gouttebarge et al., 2021). It is a self-assessment screening instrument containing 12 questionnaires designed to identify individuals at risk of suffering from certain mental health problems (Gouttebarge et al., 2021). It consists of a step-wise approach, beginning with an initial triage form, the Athlete Psychological Strain Questionnaire (APSQ), which has a cut-off score of 17 (Rice et al., 2020). If the cut-off is met, a second form containing 6 questionnaires is recommended and includes: the Patient Health Questionnaire-9 (PHQ-9), the Brief Eating Disorder in Athletes Questionnaire (BEDA-Q), the Generalised Anxiety Disorder-7 (GAD-7), the Athlete Sleep Screening Questionnaire (ASSQ), the adjusted form of the Cutting Down, Annoyance by Criticism, Guilty Feeling, and Eye-openers Adapted to Include Drugs (CAGE-AID) and the Alcohol Use Disorders Identification Test Consumption (AUDIT-C) (Gouttebarge et al., 2021). The third step comprises recommendations for clinical assessment, management of those unwell, referral to a mental health specialist and supplementary questionnaires testing for other mental health problems (such as gambling, PTSD, psychosis, etc.) (Gouttebarge et al., 2021). The English SMHAT-1 has been validated in different studies (Rice et al., 2020; Taylor et al., 2023). Having a triage step which decides the need for further evaluation, the SMHAT-1’s accuracy relies on the APSQ’s sensitivity.

The APSQ consists of ten Likert-scale type questions that evaluate the general psychological strain of the subject, considering sport-specific stressors (Rice et al., 2020). It has been translated into different languages and tested in various cultural settings (Ojio et al., 2023; Sore et al., 2024; Lma et al., 2022). The majority of the literature reported good internal consistency (Gouttebarge et al., 2021; Rice et al., 2020; Sore et al., 2024; Lma et al., 2022; García-Rubio et al., 2025) and test–retest reliability (Lma et al., 2022; Alhowimel et al., 2023), an appropriate prediction of anxiety and depression symptoms (Gouttebarge et al., 2021; Ojio et al., 2023; Shannon et al., 2024), convergent or divergent validity with validated tools (Lma et al., 2022; Ojio et al., 2021; Azadi et al., 2024), and a three-factor structure: External Coping, Self-Regulation and Performance (Lma et al., 2022; Shannon et al., 2024; Azadi et al., 2024; Waleriańczyk et al., 2024). Other results showed a different factor structure (Sore et al., 2024; Ojio et al., 2021). The APSQ’s poor ability to identify frequent mental health problems, such as eating disorders, was highlighted in some studies (Gouttebarge et al., 2021; Ojio et al., 2023; Anderson et al., 2023), alongside a high rate of false-positive and false-negative results for certain mental health problems (Taylor et al., 2023; Waleriańczyk et al., 2024). Some authors even mention that other questionnaires or different approaches might be a better practice (Waleriańczyk et al., 2024; Anderson et al., 2023). The optimal cut-off score, with an appropriate Youden index, has different values in some publications: 20 (Rice et al., 2020) or higher values (Shannon et al., 2024). Discordant results might be linked to cultural factors and reluctance to disclose mental health problems, which is often seen in athletes (Bär and Markser, 2013; Rice et al., 2016; Zhou et al., 2019; Lee et al., 2026).

Cultural influences can impact the general psychological health, support-seeking practices (Lee et al., 2026; Schinke et al., 2025), and the validity of mental health questionnaires or screening instruments (Anderson et al., 2025; Sousa and Rojjanasrirat, 2011). To our knowledge, the validity of the Romanian APSQ (APSQ-Ro) has not been published yet. To ensure its psychometric properties are similar to those of the original version, our aim was to validate the APSQ-Ro in a representative sample. Identification of a valid and reliable screening tool could improve the early detection of mental health struggles and facilitate access to mental health support for those in need. Additionally, we analysed whether there are any differences between the scores of those who are undergoing psychotherapy/counselling and those who do not receive mental health support. Psychotherapy is not commonly sought after by the athletic population (Reardon et al., 2019; Stillman et al., 2019) despite its known positive effects (Stillman et al., 2019; Rice et al., 2016). We also explored the correlation between having had a medical condition in the past 12 months, the medical decision of “ineligible for training and competitions” and the APSQ score, while accounting for a cofounding factor as part of a supplementary analysis.

## 2 Materials and methods

### 2.1 Participants and recruitment

Participants were enrolled at the National Institute of Sports Medicine in Bucharest, Romania. This is the institution where the majority of top-performing athletes from Romanian teams come for the mandatory pre-participation examination, every 6 months. One of the investigators was present during the recruitment phase. It was made clear to athletes that the study was separate from the evaluations included in the pre-participation examination and that the responses would not affect the final eligibility conclusion. The recruitment strategy was designed to enhance the credibility of the study, address potential concerns, and ensure data confidentiality in order to maximise the likelihood of accurate responses. To avoid selection bias, we did not recruit from certain types of sports. However, we only included elite athletes for whom SMHAT-1 was designed. The inclusion criteria were: 18 years old or above (due to legal consent requirements), athletes from the Romanian national and Olympic teams (to meet the ‘elite’ requirement), paralympic athletes (if present), Romanian language knowledge, informed consent, and access to an electronic device in order to complete the questionnaire. The exclusion criteria were: Paralympic athletes with visual, auditory or cognitive impairments or those who cannot type, those who did not meet the inclusion criteria and those who did not complete all the questions. The study was approved by the Ethics Committee of Carol Davila University of Medicine and Pharmacy (04.04.2025/7635) and the director of the Institute (22.04.2025/1360). The study was registered on OSF: https://doi.org/10.17605/OSF.IO/FPX4C.

### 2.2 Study design

The study was designed as a cross-sectional study and therefore followed the Strengthening the Reporting of Observational Studies in Epidemiology (STROBE) guidelines (von Elm et al., 2008). A single measure was applied to each participant. Participants had to give their consent to participate in this study by choosing the appropriate box. The questions were delivered via Google Forms and shared through a QR code. Results were anonymous to increase the chances of having accurate responses.

### 2.3 Materials

The initial set of 10 questions collected socio-demographic data and information about potential risk/protective factors, which can be found in Supplementary material S1 (the English version). The second set of questions was represented by the APSQ questionnaire. Consistent with other studies (Anderson et al., 2025; Waleriańczyk et al., 2024; Anderson et al., 2023), participants were invited to complete the battery of questionnaires from the second form of SMHAT-1, regardless of the APSQ score, in order to test the sensitivity of APSQ to detect common mental health problems. These consisted of: the General Anxiety Disorder - 7 (GAD-7) designed to assess anxiety symptoms, the Patient Health Questionnaire - 9 (PHQ-9) which assesses depressive symptoms, the Brief Eating Disorder in Athletes Questionnaire (BEDA-Q) for disordered eating, the Alcohol Use Disorders Identification Test Consumption (AUDIT-C) for alcohol misuse, the Athlete Sleep Screening Questionnaire (ASSQ) for sleep disturbances and the SMHAT-1’s version of the Cutting Down, Annoyance by Criticism, Guilty Feeling, and Eye-openers Adapted to Include Drugs (CAGE-AID) for drugs use (Gouttebarge et al., 2021). For ethical reasons, item 9 of the PHQ-9, which enquires about suicidal ideation, was not retained. As data collection was anonymous, it was not possible to provide an appropriate emergency response for participants with a positive screen. The exclusion of question 9 was performed in other similar studies (Shannon et al., 2024). We did not include the rest of the questionnaires from the third step of SMHAT-1, as seen in other studies (Taylor et al., 2023), to avoid making the examination overly demanding for athletes. This approach has been reported in other studies (Ojio et al., 2023; Waleriańczyk et al., 2024; Anderson et al., 2023). To perform divergent validity testing, the athletes had to complete a final questionnaire, the World Health Organisation – Five Well-Being Index (WHO–5) (WHO, 2024). The divergent validity comparison of APSQ with the WHO–5 was previously documented in another study (Ojio et al., 2021). The WHO-5 is a self-assessment questionnaire of psychological well-being that has been validated in different studies (Sischka et al., 2020; Nylén-Eriksen et al., 2022). The reason for choosing this questionnaire and not others that were used in APSQ validation studies, such as Kessler-10 (Rice et al., 2020; Azadi et al., 2024; Rice et al., 2020; Kessler et al., 2002), the Mental Health Continuum – Short Form (MHC-SF) (Shannon et al., 2024), The Warwick-Edinburgh Mental Wellbeing Scales (Rice et al., 2020; Rice et al., 2020) or The Ryff Mental Well-Being Scale (RMWBS) (Azadi et al., 2024) was due to the lack of validated Romanian versions. The Romanian version of WHO-5 was retrieved from the WHO website (WHO, 2024) and validated in a study that included Romanian participants (Preoteasa and Preoteasa, 2015). The Depression, Anxiety and Stress Scale (DASS-21) was not selected due to its length, even though it was used to validate the Turkish version of APSQ (Lma et al., 2022), and the Romanian version of it exists and was validated (Zanon et al., 2020).

### 2.4 Translation

The Romanian version of the SMHAT-1 was translated by a group of researchers and was published on the IOC website. As no publication was found regarding the translation process, we decided to carry out our own translation and compare the result with the one available on the IOC website. Similar to other studies that have translated questionnaires, we followed a structured process (Sore et al., 2024; Alhowimel et al., 2023; Ojio et al., 2021; Guillemin et al., 1993): translation from English to Romanian, by an independent researcher, proficient in both languages (TD), revision of the translation by another researcher, proficient in both languages and with Sports Medicine expertise (AS), with no disagreements observed regarding the items, and reverse translation from Romanian to English conducted by one researcher, expert in Public Health and proficient in both languages (FF). The comparison between the original APSQ and the one that resulted from reverse translation was performed by an expert in Sports Psychology and a native English speaker (OR). No amendments were considered useful to our Romanian translation. All authors evaluated the differences between the published Romanian version and the one that resulted from our translation. The differences were not deemed significant, so no changes were made. Therefore, the version used in this study was the one publicly available on the IOC website and attributed to the group of researchers who performed and reported the translation (National Olympic Committee, n.d.). We also sought permission (via e-mail) from Prof. Dr. Vincent Gouttebarge to perform the validation, who confirmed no approval was required, as well as from the authors who produced the Romanian translation.

### 2.5 Statistical analysis

The formal analysis was undertaken using JASP (version 0.95.4). The validation analysis consisted of internal consistency testing, exploratory and confirmatory factor analysis (EFA, CFA), divergent validity, and diagnostic accuracy of the established cut-off value of 17 for detecting common mental health problems. Person’s correlation coefficients were calculated based on the total scores of APSQ and WHO-5.

#### 2.5.1 Internal consistency testing

The Cronbach’s alpha value is meant to assess the internal consistency of a questionnaire and, therefore, the correlation between items (Tavakol and Dennick, 2011). Scores can range between 0 and 1, with higher values suggesting a stronger correlation and ability to detect the same aspect (Tavakol and Dennick, 2011). Values ≥ 0.7 are associated with an acceptable internal consistency of the scale (Tavakol and Dennick, 2011). Values ≥ 0.9 are considered excellent (Tavakol and Dennick, 2011).

#### 2.5.2 Exploratory and confirmatory analysis

To reveal the factor structure of the APSQ-Ro, we performed an Exploratory Factor Analysis (EFA), consistent with previous studies (Rice et al., 2020; Sore et al., 2024; Ojio et al., 2021). The sample size was adequate for the analysis (SPSS Analysis, n.d.). We performed the Kaiser-Meyer-Olkin and Bartlett’s test to ensure the items were factorable, we explored the factor loading of each item, analysed the scree plot, and conducted a promax oblique rotation (Rice et al., 2020; Sore et al., 2024; SPSS Analysis, n.d.). Kaiser-Meyer-Olkin test values above 0.80 indicate a high level of sampling adequacy, supporting the suitability of the data for factor analysis (Statistics Menu, n.d.). Bartlett’s Test of Sphericity assesses the suitability of the data for factor analysis by testing the null hypothesis that there are no correlations among the items. A *p*-value less than 0.05 indicates that the items are sufficiently interrelated and thus suitable for factor extraction (Statistics Menu, n.d.). Consistent with the confirmatory models used in other validation studies, we computed several model fit indices, including the Comparative Fit Index (CFI), the Tucker-Lewis Index (TLI), Goodness-of-fit (GOF), the Root Mean Squared Error of Approximation (RMSEA), and the Standardised Root Mean Squared Residual (SRMR) (Rice et al., 2020; Lma et al., 2022; Ojio et al., 2021; Waleriańczyk et al., 2024). Values indicating an adequate fit were as follows: CFI and TLI ≥ 0.90–0.95 (acceptable fit)/≥0.97 (good fit), GOF ≥ 0.90 (acceptable fit)/ ≥ 0.95 (good fit), RMSEA and SRMR ≤ 0.05 (good fit)/ ≤0.08 (adequate fit) (Schermelleh-Engel et al., 2003; Marsh et al., 2004).

#### 2.5.3 Divergent validity testing between the APSQ and the WHO-5

Divergent validity was calculated using Pearson’s correlation coefficients, in line with other research (Gouttebarge et al., 2021; Rice et al., 2020; Lma et al., 2022; Shannon et al., 2024; Ojio et al., 2021; Azadi et al., 2024).

#### 2.5.4 Diagnostic metrics of the APSQ cut-off

The sensitivity, specificity, positive predictive value and negative predictive value of the predefined cut-off score of 17, benchmarked against SMHAT-1 Step 2, were calculated, along with the area under the curve (AUC) for this threshold (Rice et al., 2020; Shannon et al., 2024; Waleriańczyk et al., 2024; Rice et al., 2020). Additionally, we calculated the overall AUC for the full range of APSQ scores against SMHAT-1 Step 2.

#### 2.5.5 Linear regression – Supplementary material S2

To investigate if there is a statistically significant difference in the APSQ score of those undergoing psychotherapy and those who never had, whilst controlling for a potential confounding factor (negative experience with an impact on the psychological well-being), we used linear regression. To examine differences in APSQ scores between individuals who had a medical condition in the past 12 months and were found ineligible for training and competition following their pre-participation examination, while taking into account the same confounding factor, we applied linear regression. The results of this investigation can be found in Supplementary material S2.

### 2.6 Pilot study

We performed a pilot study aiming to include 15 athletes. Of 16 athletes recruited, 14 completed the online questionnaires and then provided feedback. The pilot study was conducted on the 23rd of April 2025. One of the main investigators was present during the completion of the questions. Feedback was shared during a brief, unstructured interview to assess the level of comprehension of the questionnaire. The brief interview did not follow a structured template, and it was similar to an open conversation about the clarity of the questions, the length of the questionnaires, the issues addressed and potential inconveniences caused by the test. The background questions included in the first section comprised more items than those included in the final version. In the main study, we retained only those questions considered to be more important and excluded others in order to reduce the time required for completion. The results of this pilot test were not included in the formal analysis. The aim of this pilot study was to unveil potential aspects that might affect the main study. Although the feedback was not formally recorded, the general consensus among the athletes was that the questions related to illicit substances were not relevant to them, that these questions lacked all the necessary response options, and that one question in the alcohol consumption section was insufficiently precise.

## 3 Results

The statistical analysis was conducted on 206 participants in October 2025. The minimum sample size needed to validate a questionnaire of ten items was deemed to be approximately one hundred (ten participants per question) (MacCallum et al., 1999; Mundfrom et al., 2005).

### 3.1 Descriptive statistics

The demographic questions were meant to assess the representativeness of the sample. 206 athletes completed the questionnaires. The gender distribution was perfectly balanced, with exactly 103 males and 103 females, and no participants identified with another category. The majority of athletes assigned a gender at birth currently identify with that same gender, with only one athlete identifying differently. The age interval spanned from 18 to 39 years old, with a mean of 21.78 years old (SD = 4.12). The majority of athletes fell withing the 18–20 age group (53% - 109 athletes), with 29% (60 athletes) belonging to the 21–25 years old group, 14% (28 athletes) to the 26–30 years old group, 3% (6 athletes) to the 31–35 years group and only 1% (3 athletes) to the over 36 years old group. We included athletes from various sports disciplines: rowing - 45 athletes, combat sports (Greco-Roman wrestling, freestyle wrestling, taekwondo, Ju jitsu, judo, karate, boxing) – 35 athletes, athletics (any type) – 24 athletes, handball – 21 athletes, football – 17 athletes, basketball – 15 athletes, skiing (alpine skiing, cross-country skiing, ski jumping) – 10 athletes, archery/shooting sport/biathlon – 10 athletes, fencing – 9 athletes, luge/bobsleigh – 4 athletes, other sports (rugby, weightlifting, snooker, skating, table tennis, gymnastics) – 16 athletes. Only 5% of the athletes belonged to a minority ethnic group, and 5% did not disclose their ethnic background. This information had the purpose of showcasing the diversity of the sample population. None of the athletes reported having a disability. 50% of the athletes have never received psychological support, while 33.5% had access to mental health support in the past. Only 7.77% currently see a therapist at least twice per month, whilst 5.83% less than twice per month. Additionally, 2.91% did not wish to disclose this information. 133 athletes out of 206 met the APSQ cut-off threshold.

### 3.2 Reliability assessment

The Cronbach’s alpha coefficient of the Romanian APSQ demonstrated good internal consistency, with a value of 0.841 (0.811–0.870). The item-rest correlation analysis for each question varied from 0.366 (for question 9), meaning an acceptable correlation, to 0.621 (for question 4), showing strong correlations.

### 3.3 Exploratory factor analysis

The Kaiser–Meyer–Olkin test revealed an overall value of 0.884, with values varying between 0.825 (item 9) and 0.900 (item 4), highlighting the adequacy of factor analysis. Moreover, the *p*-value of <0.001 resulting from Bartlett’s test of sphericity indicated the suitability of the data for factor analysis. In accordance with the Kaiser criterion (Exploratory Factor Analysis, n.d.), we only included factors with eigenvalues above 1. Both the scree plot and the parallel analysis pointed towards a one-factor solution. Accordingly, the scale was treated as unidimensional. Nine items demonstrated significant loadings on this factorial structure, whereas Item 9 (“I needed alcohol or other substances to relax.”) did not load sufficiently to be included in the structure. Items’ loadings ranged from 0.459 (Q10) to 0.683 (Q4). Factor 1 included all the questions from the ‘Self-Regulation’ and ‘Performance’ domains described by Rice et al. (2020) in the original APSQ, along with Item 10 from the ‘External Coping’ domain (Rice et al., 2020). A one-factor structure was also observed in the Japanese APSQ validation study, though all items were included in that model (Ojio et al., 2023).

**Figure 1:**
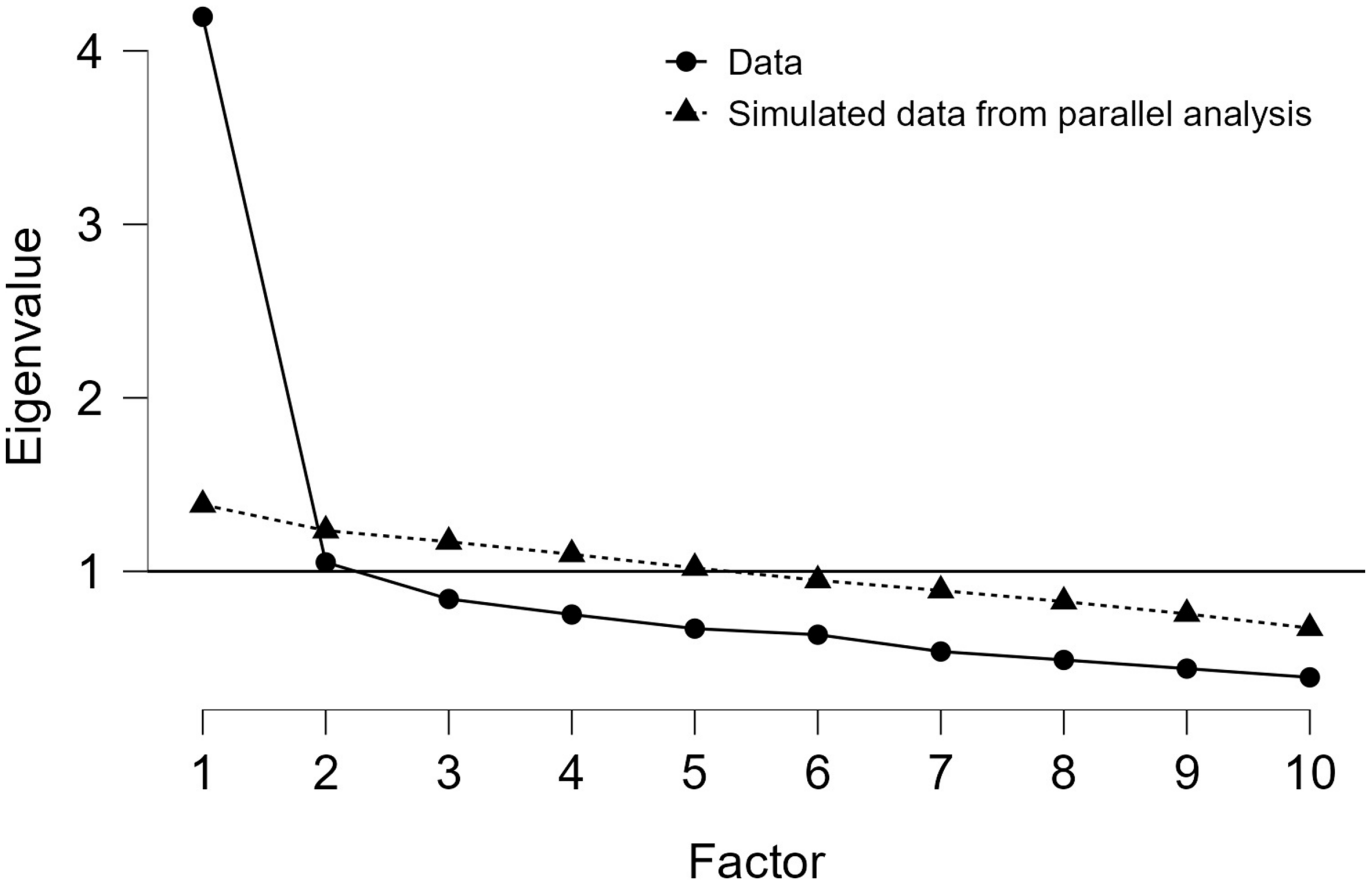
Exploratory factor analysis using parallel analysis to demonstrate a one-factor solution.

Table 1 provides the factor loadings of all APSQ question resulted from promax rotation within the exploratory factor analysis. Figure 1 represents the scree plot resulting from exploratory factor analysis.

**Table 1:** Factor loadings of all APQS questions using promax rotation (EFA).

Factor	Factor loadings
Factor 1	—
Question 1	0.626
Question 2	0.665
Question 3	0.677
Question 4	0.683
Question 5	0.581
Question 6	0.634
Question 7	0.634
Question 8	0.560
Question 10	0.459

### 3.4 Confirmatory factor analysis

The unidimensional aforementioned model was further analysed using model-fit indices. The chi-squared test for the model fit yielded a *p*-value <0.01 (*p* = 0.009; *df* = 27, *χ*^2^ = 47.428), which suggests the model does not provide a good fit to the data. However, given the sensitivity of the chi-square test to sample size, with smaller samples yielding higher *p*-values (Alavi et al., 2020), fit indices were prioritised in the interpretation of the results. The CFI, TLI above 0.90 and GOF of 0.949 values indicated an acceptable-good model fit. In addition, the RMSEA below 0.08 and the SRMR values below 0.05 confirmed the adequacy of the model fit. Standardised factor loadings, in this analysis, were modest, varying from 0.338 (Item 10) to 0.650 (Item 5), yet all loadings were statistically significant. Item 10, referring to taking usual risks, had the weakest contribution to the overall factor, albeit it remained statistically significant. This might showcase the cultural influences in responding to such topics.

Table 2 includes the values of the model fit indices resulting from confirmatory factor analysis.

**Table 2:** Confirmatory factor analysis using model fit indices.

Index/metric	Value
Comparative fit index (CFI)	0.961
Tucker-lewis index (TLI)	0.948
Goodness-of-fit index (GOF)	0.949
Root mean square error of approximation (RMSEA)	0.061 [0.030–0.089]
Standardised root mean squared residual (SRMR)	0.046

### 3.5 Divergent validity

The correlation between the APSQ-Ro and the World Health Organisation – Five Well-Being Index (WHO-5) was assessed using Pearson’s correlation coefficient. We analysed the relationship between the total score of the APSQ-Ro and the total WHO-5 score. Pearson’s *r* value of −0.594 (95% CI: −0.676 to −0.498, *p* < 0.001) indicated a significant and strong negative correlation between the two scales.

**Figure 2:**
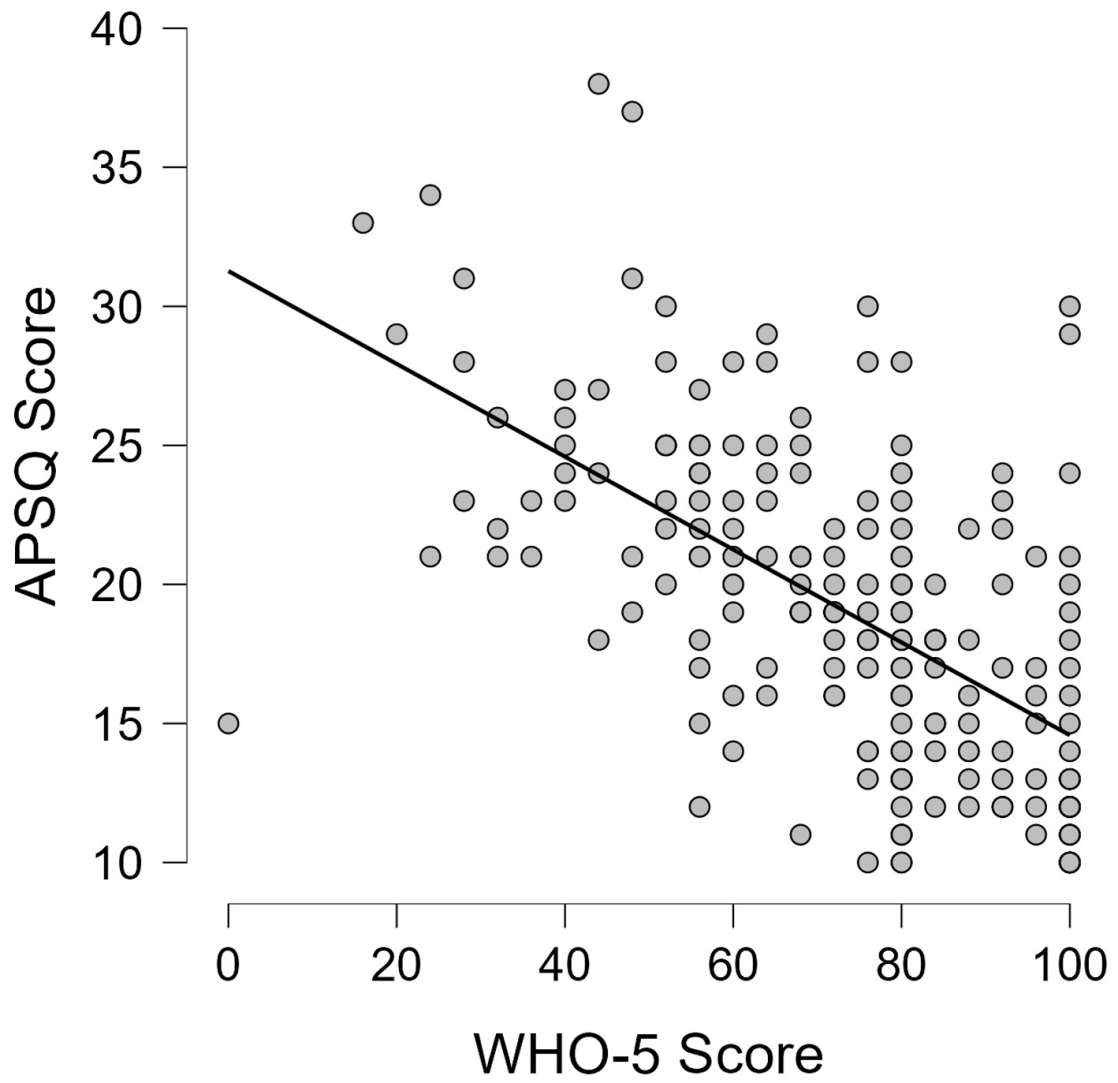
Scatter plot illustrating the association between the APSQ score and the WHO-5.

Figure 2 illustrates the scatter plot depicting the associations between the APSQ score and the WHO-5 score.

### 3.6 Performance metrics

We analysed the performance metrics of the APSQ’s predefined cut-off value of ≥17 for detecting mental health issues as classified by SMHAT-1 Step 2. For this evaluation, we generated contingency tables in JASP and manually calculated the sensitivity, specificity, positive predictive value (PPV) and negative predictive value (NPV) at the defined thresholds. We used the APSQ cut-off score and the cut-off values of each diagnostic questionnaire included in the second step of the APSQ as the gold standard. As a screening and triage tool, the APSQ is expected to demonstrate high sensitivity to identify true positive cases effectively. In addition, we examined the area under the AUC value using logistic regression between the APSQ cut-off and the corresponding cut-offs of the other questionnaires. AUC values above 0.90 typically indicate strong diagnostic performance, whereas values below 0.60 are generally considered uninformative (Shannon et al., 2024; Çorbacıoğlu and Aksel, 2023). Values falling between 0.70 and 0.90 are regarded as indicating good/fair accuracy (Çorbacıoğlu and Aksel, 2023). Furthermore, we examined the ROC curve and corresponding AUC values for the full range of APSQ scores in relation to the binary outcomes (positive/negative) of each diagnostic test, using logistic regression.

#### 3.6.1 Anxiety

The prevalence of positive anxiety screens was 9.2% (19 athletes). The APSQ threshold demonstrated a sensitivity of 100%, a specificity of 39%, a PPV of 14.28%, and a NPV of 100% for anxiety. Therefore, all participants with a positive anxiety screen had a positive APSQ result, though a positive APSQ was not a good predictor of anxiety (high rate of false positives). As a screening instrument, it is important to avoid missing potential cases. The AUC, based on logistic regression of a positive APSQ result against a positive GAD-7 result, was 0.695, indicating weak to moderate discriminative ability in distinguishing athletes with and without anxiety. This might have been caused by the low specificity. However, when examining the ROC curve for the continuous APSQ scores, the overall AUC indicated excellent discriminatory ability, with a value of 0.906.

#### 3.6.2 Depression

The overall prevalence of positive depression cases in the sample was 7.3% (15 athletes). The sensitivity of the APSQ to detect depression cases, as classified by the PHQ-9 (excluding the suicidal ideation item), reached 100%, with a specificity of 38.22%, a PPV of 11.28% and an NPV of 100%. Similar to the aforementioned analysis, this suggests that, at the selected thresholds, the APSQ identified all depression cases but yielded a high number of false positive cases. Following the removal of question 9, the cut-off score of PHQ-9 was adjusted to be 9. The AUC value of 0.691 indicated a borderline discriminative ability of the APSQ cut-off to detect depression cases, probably due to the low specificity. In addition, when analysing the ROC curve using continuous APSQ scores, the global AUC of 0.883 reflected a good overall diagnostic performance, indicating that the continuous APSQ values more accurately discriminated between athletes with and without depressive symptoms compared with the established cut-off.

#### 3.6.3 Sleep disturbance

The sensitivity of the APSQ threshold to detect sleep problems, according to the ASSQ questionnaire, was 85.71%, the specificity was 36.98%, the PPV was 9.02% and the NPC was 97.26%. Therefore, APSQ effectively detected most true positive cases but also produced a relatively high number of false positives. The AUC for the APSQ (cut-off = 17) against the ASSQ criterion was 0.613, demonstrating a limited ability to distinguish between athletes with and without sleep problems. When considering the full range of continuous APSQ scores, the overall AUC was 0.718, suggesting an acceptable diagnostic accuracy.

The prevalence of sleep disturbances in this sample was 6.8% (14 athletes).

#### 3.6.4 Alcohol misuse

The sensitivity of the APSQ in detecting alcohol misuse (as measured by the AUDIT-C) was 78.95%, the specificity was 36.90%, the PPV was 11.28% and the NPV was 94.52%. The AUC was 0.579. The very low AUC value suggests that even though the sensitivity and the NPV values were acceptable, indicating that most athletes with alcohol misuse were identified, the APSQ threshold showed a poor discriminative accuracy to detect alcohol misuse. The low specificity value reflected the high number of false positive cases. In contrast, the overall AUC value of 0.707 was indicative of a fair discriminative ability. The prevalence of alcohol misuse cases in this sample was 9.3% (19 athletes).

#### 3.6.5 Drug(s) use

The APSQ cut-off score demonstrated very low sensitivity (62.5%), specificity (35.35%), and PPV (3.76%) values. Despite these values, the NPV value was excellent (95.89%), primarily due to the low prevalence of positive cases in the sample (3.9%). The AUC of 0.511 when assessed against the diagnosis of drug(s) misuse, as measured by CAGE-AID, indicated poor discriminative ability. The test’s overall performance metrics remained low, meaning the diagnostic accuracy in detecting drug use was not evident in this study. In accordance with this, the global AUC value was 0.608. The prevalence of CAGE-AID positive results was 3.9% (8 athletes).

#### 3.6.6 Disordered eating

The APSQ’s cut-off sensitivity to detect disordered eating, as measured by BEDA-Q, in this case, was moderate (71.56%). However, the specificity had a poor value of 43.30%. The PPV was 58.65% and the NPV was 57.53%. The AUC value at the cut-off was 0.574, indicating that the APSQ’s diagnostic performance when testing for disordered eating problems could not be demonstrated in this study. Moreover, this was supported by the 0.612 overall AUC value for the range of APSQ scores.

In this sample, the prevalence of positive BEDA-Q cases was high, with more than half of the participants scoring above the established threshold (52.9% - 109 athletes).

## 4 Discussion

The APSQ-Ro demonstrated very good internal consistency, significant divergent validity with the WHO-5, adequacy for factor analysis and a one-factor model that fits the data. Good Cronbach’s alpha values have been reported in other validation studies (Mountjoy et al., 2023; Gouttebarge et al., 2021; Shannon et al., 2024; Rice et al., 2020), alongside evidence supporting the suitability of the scale for factor analysis (Rice et al., 2020; Sore et al., 2024). A study involving non-elite athletes also showed good factor loadings for the one-factor solution, though the three-factor model showed superior model fit indices and was considered optimal (Shannon et al., 2024). In contrast, a two-factor structure was revealed in the Croatian APSQ validation study (Sore et al., 2024). Still, the most encountered structure remained the three-factor structure of the original, English APSQ, the Turkish, Persian and Polish versions (Rice et al., 2020; Lma et al., 2022; Azadi et al., 2024; Waleriańczyk et al., 2024). Variations in the factor structure may reflect cultural influences that hinder open discussion of mental health, as well as potential challenges in item interpretation (Sore et al., 2024; Zhou et al., 2019; Schinke et al., 2025). The exclusion of item 9 from the factorial structure (due to insignificant factor loading) may reflect that substance-related coping mechanisms are not necessarily representative of the psychological strain construct assessed by the APSQ. Moreover, it may suggest a low endorsement of this item among athletes. Aligned with this finding, this specific item had the lowest factor loading in the validation study of the Turkish APSQ (Lma et al., 2022). However, the Turkish factorial structure comprised three elements and the one including item 9, consisted of only two items (Lma et al., 2022). The ability of the 17-point cut-off to detect common mental health issues was quite limited, probably due to the general low specificity for all the issues tested for in SMHAT’s second step. This cut-off had borderline diagnostic abilities for anxiety and depression, approaching fair discriminative ability. It also showed good sensitivity to detect sleep disturbances. The adequate performance in detecting anxiety and depression symptoms was also documented in other validation studies (Anderson et al., 2025; Shannon et al., 2024; Waleriańczyk et al., 2024; Anderson et al., 2023). On the other hand, based on the AUC value at the 17 cut-off, this threshold did not demonstrate adequate performance in detecting sleep disturbances, alcohol misuse, drug use, or eating disorders. This low performance of the cut-off in detecting eating disorders has also been acknowledged in other studies (Gouttebarge et al., 2021; Anderson et al., 2025; Taylor et al., 2023; Waleriańczyk et al., 2024; Anderson et al., 2023), as well as the limited performance in detecting alcohol misuse (Gouttebarge et al., 2021; Anderson et al., 2025; Waleriańczyk et al., 2024). Disordered eating is particularly prevalent in this population (Reardon et al., 2019; Gwyther et al., 2024; Rice et al., 2016; Åkesdotter et al., 2020), underscoring the importance of including items that can identify potential eating-related problems in mental health screening tools. The weak screening performance in this case might have been due to several factors, including cultural barriers leading to a lack of trust among athletes in disclosing mental health issues (Lee et al., 2026; Schinke et al., 2025), inadequate comprehension of the questions, limited self-awareness regarding their emotional states, or a lack of attention when completing the questionnaire. When analysing the ROC curve, the AUC overall values, for the range of APSQ values, were good to excellent for anxiety and depression, acceptable for sleep issues and alcohol misuse and poor for drug use and disordered eating. Again, these performance metrics should be interpreted in the light of possible confounding factors or cultural influences.

While some psychometric properties of the APSQ-Ro differed in this study from other reported ones (e.g., in the factorial structure, in the diagnostic accuracy for common mental health problems), it is recommended to take into account the limitations of this study. Firstly, the sample size was smaller compared to some of the SMHAT/APSQ validation studies (Mountjoy et al., 2023; Shannon et al., 2024; Rice et al., 2020). Secondly, to our knowledge, some of the questionnaires used to assess the APSQ diagnostic ability for common mental health conditions (SMHAT-1 step 2), such as BEDA-Q, were not validated in Romanian. This might have been a potential flaw when assessing the APSQ’s sensitivity and specificity. However, they were part of the official Romanian translation, published on the IOC website and translated by a group of experts in Sports Psychology. Therefore, they were considered reliable. Additionally, cultural influences might have prevented the disclosure of mental health issues (Lee et al., 2026; Schinke et al., 2025), and some athletes might have been distracted by other factors when completing the questionnaires electronically, in the waiting room, while awaiting their pre-participation examination.

As future directions, we invite other researchers to test the APSQ-Ro validity on a larger and more diverse sample, including Paralympic athletes, and test the diagnostic accuracy against validated tools or clinical interviews. Confirmation or amendment of the factorial structure of the APSQ-Ro would also be beneficial. The validation of other APSQ translations might contribute to collating scientific evidence that the APSQ can be safely used worldwide. In addition, to our knowledge, the acceptability of the APSQ has not yet been tested within athletes or mental health practitioners or physicians. Acceptability represents an important aspect when implementing a screening tool and deserves attention (Sekhon et al., 2022). Furthermore, it is worth noting that a psychometric validation study may not fully reflect the real-word effectiveness of a screening questionnaire. Utilising this tool in a day-to-day practice may yield different or additional outcomes. Moreover, cultural, organisational and systemic factors may influence how the tool is applied in practice and its implementation results; therefore, further cross-national research is needed and highly recommended to obtain a broader perspective on the effectiveness of this translated version as well as other translations.

## 5 Conclusion

This validation study lays the ground for further studies concerning the psychometric properties of the APSQ-Ro and other translated APSQ versions. Drawing attention to the validity of the APSQ is of paramount importance since it decides whether the athletes are further screened for mental health problems or not. The findings of this study are intended to inform clinical practice in mental health screening and to guide future research on this topic and the development of new screening tools.

## Data Availability

The raw data supporting the conclusions of this article will be made available by the authors, without undue reservation.
