# Supplementary material for: Global athlete mental health screening: cross-cultural validity of the athlete psychological strain questionnaire
Source: Front Psychol. 2026 Mar 12;17:1761749. doi: 10.3389/fpsyg.2026.1761749 (PMC13017933; doi:10.3389/fpsyg.2026.1761749)
Supplement: Supplementary file 1 [file Supplementary_file_1.docx]

Supplementary Material 1 – Additional Questions

Survey – first step

*The following questions are meant to gather general information relevant to this study. You are invited to complete the subsequent questions by choosing A SINGLE answer that best describes your situation.*

*Thank you for your time!*

1. What gender were you assigned at birth?
2. Female
3. Male
4. I prefer not to declare
5. What gender do you currently identify with?
6. Female
7. Male
8. Non-binary
9. Transgender
10. Any other options
11. I prefer not to declare
12. How old are you?

____________________________ years old

1. Do you belong to an ethnic minority group in Romania?
2. Yes
3. No
4. I prefer not to declare
5. What sport do you practice?

_________________________________________________________________________

1. Have you experienced any medical conditions in the last 12 months?
2. No
3. Yes, with consequences on my performance
4. Yes, without consequences on my performance
5. Have you received in the last 12 months an ‘ineligible for training and competing’ result following a pre-participation examination?
6. Yes
7. No
8. Have you ever benefited from mental health support through psychotherapy/counselling?
9. Yes, I currently see a therapist at least twice per month
10. Yes, I currently see a therapist, but less than twice per month
11. I did in the past, but not at the moment
12. No, I have never tried that
13. I prefer not to answer
14. Have you recently experienced an unpleasant event in your personal life that affected your emotional well-being?
15. Yes
16. No
17. I prefer not to declare
18. Do you suffer from any disability?
19. Yes – I compete in para-athletic competitions
20. No
21. I prefer not to disclose
