# Supplementary material for: Global athlete mental health screening: cross-cultural validity of the athlete psychological strain questionnaire
Source: Front Psychol. 2026 Mar 12;17:1761749. doi: 10.3389/fpsyg.2026.1761749 (PMC13017933; doi:10.3389/fpsyg.2026.1761749)
Supplement: Supplementary file 2 [file Supplementary_file_2.docx]

Supplementary Material 2

# Supplementary Data

**3.7. Correlations with other factors**

**3.7.1. Psychotherapy/counselling**

Using linear regression, we analysed the correlations between the APSQ score and psychotherapy/ counselling support, while accounting for a potential confounder: an unpleasant emotional event. Although our hypothesis relied on the fact that mental health support could have been reflected in the APSQ scores, the results in this case were not conclusive. While the overall model, including the psychotherapy support at present (regardless of how many times per month) or in the past, and the unpleasant event resulted in a significant p-value (<0.001), the p-value linked to psychotherapy was not statistically significant (p = 0.204, unstandardised coefficient = 1.015). The only significant predictor was the unpleasant emotional event (p <0.001, unstandardised coefficient 5.078).

Although in this case we could not demonstrate the potential positive effect of psychotherapy on well-being, this does not mean that a correlation does not exist. This outcome might have been affected by the reluctance to disclose previous mental health support, by the imprecision in measuring the effect of psychotherapy, or by other confounding factors not accounted for in this study. We did not consider the quality of psychotherapy, the length of therapy, nor did we differentiate between those currently undergoing therapy who may have already benefited from it and those who are just starting.

**3.7.2. Medical condition and ‘ineligible’ for training and competitions**

Similar to the previously mentioned statistical analysis, we investigated the correlations between the APSQ score, the presence of a medical condition in the last 12 months and the presence of a negative pre-participation examination result (ineligible for training and competitions in the last 12 months), while accounting for the same confounding factor (unpleasant event). The linear regression showed that the two explored factors - previous medical condition and a negative pre-participation examination result - did not have a significant effect on the APSQ score: p-value for the previous medical condition was 0.185, and for the negative pre-participation examination result, 0.271. In line with the aforementioned results, the confounding factor (the presence of an unpleasant personal event) had a statistically significant (p < 0.001) impact on the APSQ score (unstandardised coefficient 4.582).
